# The evolving landscape of SARS-CoV-2 vaccination in Mexico: real-world evidence in Mexican pensioners

**DOI:** 10.1016/j.lana.2023.100624

**Published:** 2023-10-31

**Authors:** Neftali Eduardo Antonio-Villa, Omar Yaxmehen Bello-Chavolla, Carlos A. Fermín-Martínez, Luisa Fernández-Chirino, Daniel Ramírez-García

**Affiliations:** a Departamento de Endocrinología, Instituto Nacional de Cardiología Ignacio Chávez, Mexico City, Mexico; b Research Division, Instituto Nacional de Geriatría, Mexico City, Mexico; c MD/PhD (PECEM) Program, Facultad de Medicina, Universidad Nacional Autónoma de México, Mexico City, Mexico; d Clinical Trial Service Unit & Epidemiological Studies Unit, Nuffield Department of Population Health, University of Oxford, Oxford, UK; e Facultad de Medicina, Universidad Nacional Autónoma de México, Mexico City, Mexico

The COVID-19 pandemic proved the resilience of health systems in managing a health crisis worldwide. In this sense, implementing large-scale vaccination programs was proposed as a fundamental strategy to restrict infections and deaths caused by SARS-CoV-2 infections.^1^ Within this context, low-and-middle-income countries (LMICs) faced two main challenges in implementing vaccine programs: 1) to secure enough vaccines for vulnerable populations who could experience a higher risk of adverse COVID-19 outcomes and 2) to ensure the means to continuously distribute and apply perishable vaccines in large territories.^2^

Mexico implemented a comprehensive vaccination policy encompassing up to 15 vaccines to cover almost 160 million inhabitants.^3^ Between January 2021 and October 2023, the Mexican vaccination policy administered nearly 222 million vaccine doses.^4^ This extensive coverage naturally raised a pressing inquiry: how effective was this preventive strategy in real-world scenarios? Preliminary outcomes indicated that the Mexican vaccination program showcased overall good protection rates against COVID-19 adverse outcomes.^5^, ^6^, ^7^ Yet, the vaccine's effectiveness in specific populations in Mexico remained an unsolved question.

In this issue of *The Lancet Regional Health—Americas*, Hernandez-Avila and Ortiz-Brizuela et al. performed a nationwide nested test-negative design to evaluate the effectiveness of five SARS-CoV-2 vaccines (BNT162b2, AZD1222, CoronaVac, Gam-COVID-Vac, and Ad5-nCoV) in Mexican pensioners covered by the Mexican Institute of Social Security.^8^ Using data from 28,272 individuals tested for SARS-CoV-2 infection between April and November 2021, they evaluated the effectiveness of these vaccines against symptomatic infection, hospitalization, a composite of severe disease, and death attributable to COVID-19. The authors report that all implemented COVID-19 vaccines displayed adjusted effectiveness rates of 56.3% for symptomatic SARS-CoV-2 infection, 75.3% for hospitalization, 79.7% for severe COVID-19, and 79.8% against death. They demonstrated that BNT162b2 and Gam-COVID-Vac were the most effective vaccines in preventing adverse and lethal outcomes. Furthermore, when the authors stratified by the time since vaccine application and by sex, they reported a slight decline in effectiveness after 141 days, especially in men. The main strengths of this study lie in its design, a well-defined nested cohort from the largest social security institution cross-linking data from five robust administrative and clinical databases, and a robust statistical analysis endorsed by a test-negative design which supports the authors' results. The results proposed by the authors are consistent with results from other regions of the world, although they do not consider the influence of vaccine boosters, hybrid immunity, or reinfections, which could also influence vaccine effectiveness in the long-run. Furthermore, the analyses presented by the authors convers only pensioners affiliated to social security, a population which has access to medical care and thus, does not face the high burden of treatment cost associated with severe COVID-19 as uninsured population. Therefore, this population may on average have better outcomes compared to older adults without insurement, as shown in previous research.^9^ The impact of vaccine effectiveness in uninsured population remains unexplored and should be investigated to plan and guide future vaccine rollouts to prioritize booster allocation.

The results from this study fill an important gap in Mexico, as they contribute to understanding the real-world effectiveness of a multi-vaccine program for pensioners. This subset of the population has not only been proven to face a higher risk of adverse outcomes due to increased age and comorbid conditions but also endures social disadvantages and economic vulnerability, increasing the risk of severe forms of COVID-19.^9^ The authors showed that pensioners living in more marginalized areas experienced reduced vaccine effectiveness against COVID-19 mortality. The findings highlight the challenges of implementing a multi-vaccination program in a country like Mexico, where logistical obstacles, such as maintaining the cold chain, ensuring sufficient healthcare personnel for administration and distribution, and managing rising demand in densely populated areas, are crucial for ensuring equitable vaccination access. Furthermore, these findings highlight the need for context-specific policies to promote COVID-19 vaccination, particularly with the need for subsequent bivalent vaccine boosters that have been shown with the spread of the Omicron SARS-CoV-2 variant, and which will require updating current COVID-19 vaccination programs in LMICs to ensure booster coverage to ensure further protection as future SARS-CoV-2 variants emerge.^10^ The encouraging results from Mexico's COVID-19 vaccination policies also highlight the need to continually monitor and improve vaccination programs with the evolving landscape of SARS-CoV-2 as it transitions into endemicity.

While more research is still needed to gauge the effectiveness of COVID-19 vaccines, the results from this study show that successful public health policies, such as Mexico's vaccination program, are effective strategies to mitigate the impact of the emerging pandemic. However, they also show that policies still need strengthening to address context-specific challenges that perpetuate the influence of sociodemographic inequalities on the effectiveness of these interventions in one of Latin America's hardest-hit regions.

## Contributors

Each author contributed important intellectual content during manuscript drafting or revision and accepted accountability for the overall work by ensuring that questions pertaining to the accuracy or integrity of any portion of the work are appropriately investigated and resolved.

## Declaration of interests

Nothing to disclose.
